# A Novel Risk Score Based on Lipid-Related Biomarkers for Acute Coronary Syndromes: A Multicenter Machine Learning Study

**DOI:** 10.31083/RCM44578

**Published:** 2026-02-04

**Authors:** Jingjing Wan, Yinhua Luo, Yuanhong Li, Shaoqian Cai, Ting He, Ze Chen, Feifei Yan, Yingying Hu, Zhen Zhou, Qiongxin Wang, Zhibing Lu

**Affiliations:** ^1^Department of Cardiology, Zhongnan Hospital of Wuhan University; Institute of Myocardial Injury and Repair, Wuhan University, 430071 Wuhan, Hubei, China; ^2^Hubei Provincial Clinical Research Center for Cardiovascular Intervention, 430071 Wuhan, Hubei, China; ^3^Cardiovascular Disease Center, Central Hospital of Tujia and Miao Autonomous Prefecture, Hubei University of Medicine, 442000 Shiyan, Hubei, China; ^4^Department of Cardiology, China Resources & Wisco General Hospital, Wuhan University of Science and Technology, 430071 Wuhan, Hubei, China; ^5^Department of Cardiac Ultrasonography, Zhongnan Hospital of Wuhan University, 430071 Wuhan, Hubei, China

**Keywords:** acute coronary syndrome, machine learning, high-density lipoprotein cholesterol (HDL-C) ratio, triglyceride glucose index, risk stratification

## Abstract

**Background:**

This study aimed to develop and test an explainable machine learning (ML) predictive model based on lipid-related biomarkers to predict acute coronary syndrome (ACS) in hospitalized patients.

**Methods:**

A total of 10,127 consecutive hospitalized patients at three large hospitals were retrospectively studied between 2022 and 2024. ACS incidence was recorded as the primary outcome. Eight ML models were used to calculate the risk of ACS during hospitalization and to distribute patients into low-, intermediate-, and high-risk groups.

**Results:**

All patients were randomly divided into a 70% training set (n = 7088) and a 30% test set (n = 3039). ACS occurred in 1119 (15.8%) and 461 (15.2%) patients, respectively. The Light Gradient Boosting Machine (LightGBM) exhibited the best predictive performance (area under the curve, 0.829) for ACS in the training set. The final model, which included the top 10 features from the LightGBM model, including lipid-related markers and clinical features, achieved a C-index of 0.781 on the test set and demonstrated a significant ability to stratify patients into low-, intermediate-, and high-risk groups.

**Conclusion:**

We constructed a risk-stratification model based on lipid-related biomarkers derived from ML models to predict ACS in hospitalized patients, which could assist in identifying patients with high discriminatory capacity.

## 1. Introduction

Acute coronary syndrome (ACS), which is mainly triggered by the rupture, erosion, or calcified nodules of atherosclerotic plaques and results in acute myocardial ischemia, myocardial cell necrosis, and inflammation, is a leading cause of morbidity and mortality in developed and developing countries [1, 2, 3]. Increasing data have revealed that early and timely detection of ACS and the administration of effective interventions could significantly improve patient prognosis. Therefore, doctors need to prioritize the pursuit of user-friendly biomarkers for the prompt detection of those at risk of ACS. Several risk scores, such as the Global Registry of Acute Coronary Events (GRACE) and thrombolysis in myocardial infarction (TIMI) scores, have been developed mainly for Caucasian populations with chest pain. However, these scores vary in performance, especially among the Chinese population, and might perform better in identifying patients with ACS at high risk of major adverse cardiovascular events [4, 5, 6]. Moreover, few studies have focused on the risk of ACS in hospitalized patients, regardless of whether they have chest pain.

Dyslipidemia is a notable independent risk factor for the onset and advancement of ACS and has been acknowledged as a focal point for therapeutic intervention [7, 8, 9]. Unlike the widely accepted traditional lipid indicators, a growing number of studies have found that some lipid-related biomarkers, including residual cholesterol (RC), triglyceride-glucose (TyG) index, and uric acid–to–high-density lipoprotein cholesterol (HDL-C) ratio (UHR), could serve as predictors of the incidence and prognosis of cardiovascular diseases [10, 11, 12]. However, the precision of these biomarkers or models remains relatively inadequate. Hence, there is an urgent need for further improvement to accurately stratify the risk of ACS in patients. Machine learning (ML), which is gaining prominence in various fields, can overcome these limitations [13, 14]. In recent years, several ML models have been developed to identify individuals at risk for ACS and acute myocardial infarction [15, 16]. However, most studies rely on bedside electrocardiogram detection or cardiac troponin concentrations, and some models are primarily designed based on lipid-related biomarkers. Therefore, this study aimed to develop a risk score to identify ACS risk groups in hospitalized patients using ML methods and visually interpret the model using SHapley Additive exPlanations (SHAP) methods to assist clinicians in identifying and managing high-risk groups.

## 2. Materials and Methods

### 2.1 Study Population

This retrospective multicenter study enrolled patients hospitalized in the Department of Cardiology from January 2022 to January 2024 at Zhongnan Hospital of Wuhan University, Enshi Tujia and Miao Autonomous Prefecture Central Hospital, and China Resources and Wisco General Hospital. These hospitals attract patients from diverse regions, reflecting a broad spectrum of patient characteristics and treatment preferences, and bolstering the geographic representativeness of the study.

**Fig. 1.**
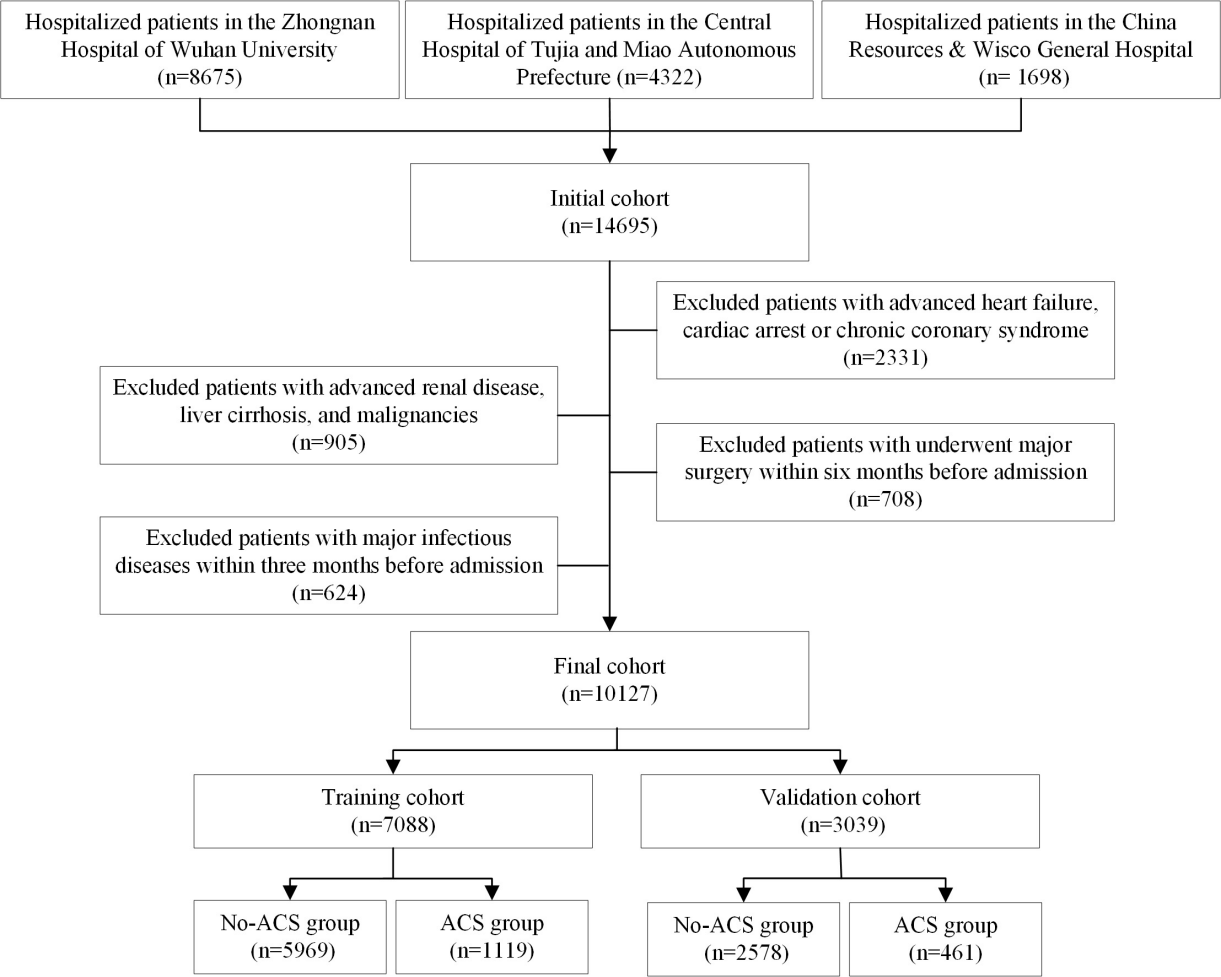
**The flowchart of this study**. ACS, acute coronary syndrome.

The inclusion criteria were adult patients admitted to the Department of Cardiology in these hospitals with complete clinical, laboratory, and imaging data. The exclusion criteria were as follows: (1) advanced heart failure (New York Heart Association functional class III or IV), history of cardiac arrest, or chronic coronary syndrome; (2) severe systemic diseases affecting lipid metabolism, such as advanced renal disease, liver cirrhosis, or malignancies; (3) major surgery within six months before admission; and (4) major infectious diseases within three months before admission. Finally, the study included 10,127 patients from the Chinese Han population (Fig. 1). To create and validate the models, all data were randomly partitioned into a 70% training set (n = 7088) and 30% test set (n = 3039).

This study was approved by the Medical Ethics Committee of Zhongnan Hospital of Wuhan University (2021053), the Medical Ethics Committee of China Resources Wisco General Hospital (CRWG2024R031J), and the Ethics Committee of Enshi Central Hospital (2024-052-01). This study complies with the Declaration of Helsinki.

### 2.2 Data Collection

Relevant clinical variables were retrospectively gathered from patient records, including demographic details (age, sex, body mass index), comorbidities (hypertension, diabetes), and initial laboratory results within 48 hours of hospital admission. The comorbidities of all patients were encoded using the International Classification of Diseases, Ninth Revision and exported from the electronic health record system. Because myocardial marker examinations were not conducted within 48 hours of hospital admission, we did not include cardiac biomarkers (brain natriuretic peptide, cardiac troponin I, and creatine kinase-MB).

Fasting blood samples were collected from each patient and analyzed using standard hospital assays. Lipid traits were analyzed using a Beckman Coulter AU5800 (Beckman Coulter Inc., Brea, CA, USA).

### 2.3 Definition of Lipid-Related Biomarkers 

RC (mmol/L) was calculated as total cholesterol minus the sum of the HDL-C and low-density lipoprotein cholesterol (LDL-C) levels [17]. The TyG index was calculated as ln (triglycerides × plasma glucose/2) [18]. The UHR was calculated as the ratio of uric acid to HDL-C [19].

### 2.4 Primary Outcome

The primary outcome of this study was the diagnosis of ACS during hospital admission, based on the 2014 guidelines of the American College of Cardiology and the American Heart Association, as determined by typical chest pain symptoms, electrocardiogram changes, and elevated cardiac biomarkers such as high-sensitivity troponin [20].

### 2.5 Model Construction

First, we manually selected potential variables, guided by clinical knowledge, previous studies, and accessibility. To provide a simple, easy-to-use, and relatively accurate diagnostic model for all clinicians to stratify patient risk, we only included common clinical variables. In the end, our structured database comprised 27 clinical variables, referred to as “features” for candidate predictors.

We used eight ML algorithms: Decision Tree, Random Forest, Extreme Gradient Boosting (XGBoost), Multilayer Perceptron, Light Gradient Boosting Machine (LightGBM), Support Vector Machine, Elastic Network (ENet), and Logistic Regression [21, 22, 23].

### 2.6 Machine Learning Interpretation Tools

We used SHAP analysis to identify potentially important factors, and then tested whether the models could sufficiently predict the outcomes by choosing fewer variables. During the model generation process, the features that had the least influence on the outcome were gradually deleted, and the models were repeatedly rebuilt with smaller sets of features. This iterative removal proceeded until a considerable drop in the model performance was observed. This repeated procedure refined our models to focus on the most predictive factors. Finally, a model risk score based on lipid-related biomarkers was used to categorize the patients into three distinct risk groups: low, intermediate, and high risk of ACS.

### 2.7 Statistical Analysis

Statistical analyses were conducted using R (version 4.3.2, R Foundation for Statistical Computing, Vienna, Austria) and Python (version 3.11, Python Software Foundation, Wilmington, DE, USA). Various evaluation metrics such as sensitivity, specificity, F1 score, and area under the curve (AUC) were employed to comprehensively evaluate the performance of the ML models. Continuous variables are presented as mean ± standard deviation, while categorical variables are presented as counts and percentages. Receiver operating characteristic (ROC) curves and Youden index were used to determine the cutoff values of continuous variables for the risk score. Detailed information on the analysis and feature selection is provided in the **Supplementary material**. Statistical tests were 2-tailed, with *p* < 0.05 denoting significance.

## 3. Results

### 3.1 Patient Characteristics

A total of 10,127 patients were enrolled in this study. Most of the cohort was male (62.8%), and the mean age of all participants was 64.9 ± 11.1 years (range, 24–96). Hypertension was present in 63.6% of patients, and diabetes in 27.0%. Hyperuricemia was observed in 6.0% of patients, and 1701 (16.8%) were current smokers. Moreover, ACS occurred in 1580 (15.6%) patients during hospital admission.

Table 1 and **Supplementary Table 1** show the characteristics of all patients in this study, as well as the comparison of the different groups.

**Table 1.** **Baseline characteristics of the overall cohort and outcome groups**.

Characteristics		Overall (n = 10,127)	ACS (n = 1580)	No-ACS (n = 8547)	*p* value
Age, years old		64.9 ± 11.1	65.8 ± 12.2	64.7 ± 10.8	<0.001
Gender, male, n (%)		6361 (62.8)	1228 (77.7)	5133 (60.1)	<0.001
Current smoker, n (%)		1701 (16.8)	372 (23.5)	1329 (15.5)	<0.001
Comorbidities, n (%)					
	Hypertension	6438 (63.6)	1072 (67.8)	5366 (62.8)	<0.001
	Diabetes	2732 (27.0)	557 (35.3)	2175 (25.4)	<0.001
	HLP	1647 (16.3)	313 (19.8)	1334 (15.8)	0.017
	HUA	611 (6.0)	108 (6.8)	503 (5.9)	0.740
	Stroke	2691 (26.6)	498 (31.5)	2193 (25.7)	0.005
	AF	1749 (17.3)	315 (19.9)	1434 (16.8)	0.001
Laboratory results					
	WBC, ×10^9^/L	6.6 ± 2.2	8.0 ± 2.2	6.4 ± 2.1	<0.001
	Hemoglobin, g/L	137.3 ± 18.2	134.6 ± 21.8	137.8 ± 17.4	<0.001
	Platelet, ×10^9^/L	207.3 ± 50.9	211.2 ± 47.0	206.5 ± 49.7	0.005
	Lymphocytes, ×10^9^/L	1.2 (0.5, 1.7)	1.3 (0.9, 1.8)	1.2 (0.5, 1.7)	<0.001
	Monocytes, ×10^9^/L	0.5 (0.3, 1.1)	0.5 (0.4, 0.7)	0.5 (0.3, 1.2)	<0.001
	AST, U/L	20.0 (14.0, 29.0)	20.0 (15.0, 29.0)	20.0 (14.0, 29.0)	0.004
	ALT, U/L	19.0 (13.0, 28.0)	22.0 (15.0, 37.0)	19.0 (13.0, 27.0)	<0.001
	TBIL, µmol/L	11.4 (8.5, 15.1)	11.4 (8.2, 15.1)	11.4 (8.6, 15.2)	0.003
	DBIL, µmol/L	3.3 (2.4, 4.6)	3.5 (2.5, 4.8)	3.3 (2.4, 4.5)	<0.001
	Albumin, g/L	39.9 ± 4.3	38.7 ± 4.4	40.1 ± 4.2	<0.001
	BUN, umol/L	5.0 (4.2, 5.9)	5.0 (4.2, 5.8)	5.0 (4.2, 5.9)	0.005
	Creatinine, umol/L	84.6 ± 25.4	99.5 ± 26.1	81.9 ± 20.4	<0.001
	Potassium, mmol/L	4.0 ± 0.4	4.0 ± 0.4	4.0 ± 0.4	0.005
	Sodium, mmol/L	138.3 ± 3.8	138.3 ± 3.8	138.3 ± 3.7	0.756
Lipid-related biomarkers					
	RC	0.5 (0.3, 0.7)	0.5 (0.3, 0.7)	0.5 (0.3, 0.7)	<0.001
	UHR	285.4 ± 60.8	348.7 ± 65.8	273.7 ± 57.0	<0.001
	TyG	8.8 ± 0.7	9.0 ± 0.7	8.8 ± 0.7	<0.001
	Lp(a), mg/L	170.5 ± 62.8	236.3 ± 60.8	158.3 ± 52.0	<0.001

HLP, hyperlipidemia; HUA, hyperuricemia; AF, atrial fibrillation; WBC, white blood cells; AST, aspartate aminotransferase; ALT, alanine aminotransferase; TBIL, total bilirubin; DBIL, direct bilirubin; BUN, blood urea nitrogen; RC, residual cholesterol; UHR, uric acid to high-density lipoprotein-cholesterol ratio; TyG, triglyceride-to-glucose index; ACS, acute coronary syndrome; Lp(a), lipoprotein(a).

### 3.2 Performance of Different Machine Learning Models

We used seven machine learning models and one conventional logistic regression model to identify the most informative metrics associated with ACS in hospitalized patients in the training set. ROC curves were generated to objectively assess the overall performance of the prediction models. As shown in Table 2, the LightGBM model exhibited the best predictive ability (AUC = 0.829), followed by XGBoost (AUC = 0.807) and Random Forest (AUC = 0.803). The ENet model showed the worst predictive performance (AUC = 0.753). In the test set, the LightGBM model again exhibited the best predictive ability (AUC = 0.790), whereas the decision tree model showed the worst predictive performance (AUC = 0.685). Other parameters related to the predictive models, such as accuracy, sensitivity, positive predictive value, negative predictive value, and F1 score, are listed in Table 2 and **Supplementary Figs. 1,2**. Therefore, we selected LightGBM as the optimal model for risk stratification and further analysis.

**Table 2.** **Comparing the predictive performance of different models**.

Models		AUC	Accuracy	Sensibility	Specificity	F1 score	PPV	NPV
In the training set								
	Decision trees	0.759	0.693	0.656	0.700	0.401	0.288	0.917
	Random Forest	0.803	0.756	0.723	0.758	0.637	0.486	0.983
	XGBoost	0.807	0.716	0.738	0.712	0.448	0.322	0.936
	LightGBM	0.829	0.787	0.796	0.785	0.557	0.423	0.949
	SVM	0.778	0.719	0.690	0.724	0.433	0.316	0.927
	MLP	0.764	0.668	0.731	0.656	0.407	0.282	0.930
	ENet	0.753	0.638	0.769	0.614	0.398	0.269	0.935
	Logistic	0.759	0.667	0.723	0.657	0.404	0.280	0.928
In the test set								
	Decision trees	0.685	0.677	0.553	0.700	0.348	0.254	0.894
	Random Forest	0.771	0.723	0.662	0.734	0.427	0.315	0.922
	XGBoost	0.775	0.701	0.705	0.700	0.424	0.303	0.928
	LightGBM	0.790	0.740	0.683	0.750	0.436	0.335	0.931
	SVM	0.759	0.714	0.673	0.722	0.424	0.309	0.923
	MLP	0.755	0.676	0.736	0.665	0.415	0.289	0.932
	ENet	0.748	0.640	0.757	0.618	0.396	0.269	0.932
	Logistic	0.749	0.671	0.717	0.663	0.405	0.282	0.927

XGBoost, Extreme Gradient Boosting; LightGBM, Light Gradient Boosting Machine; SVM, Support Vector Machine; MLP, Multilayer Perceptron; ENet, Elastic Network; AUC, the area under the curve; PPV, positive predictive value; NPV, negative predictive value.

### 3.3 Construction and the Performance of the Risk Score for ACS

**Fig. 2.**
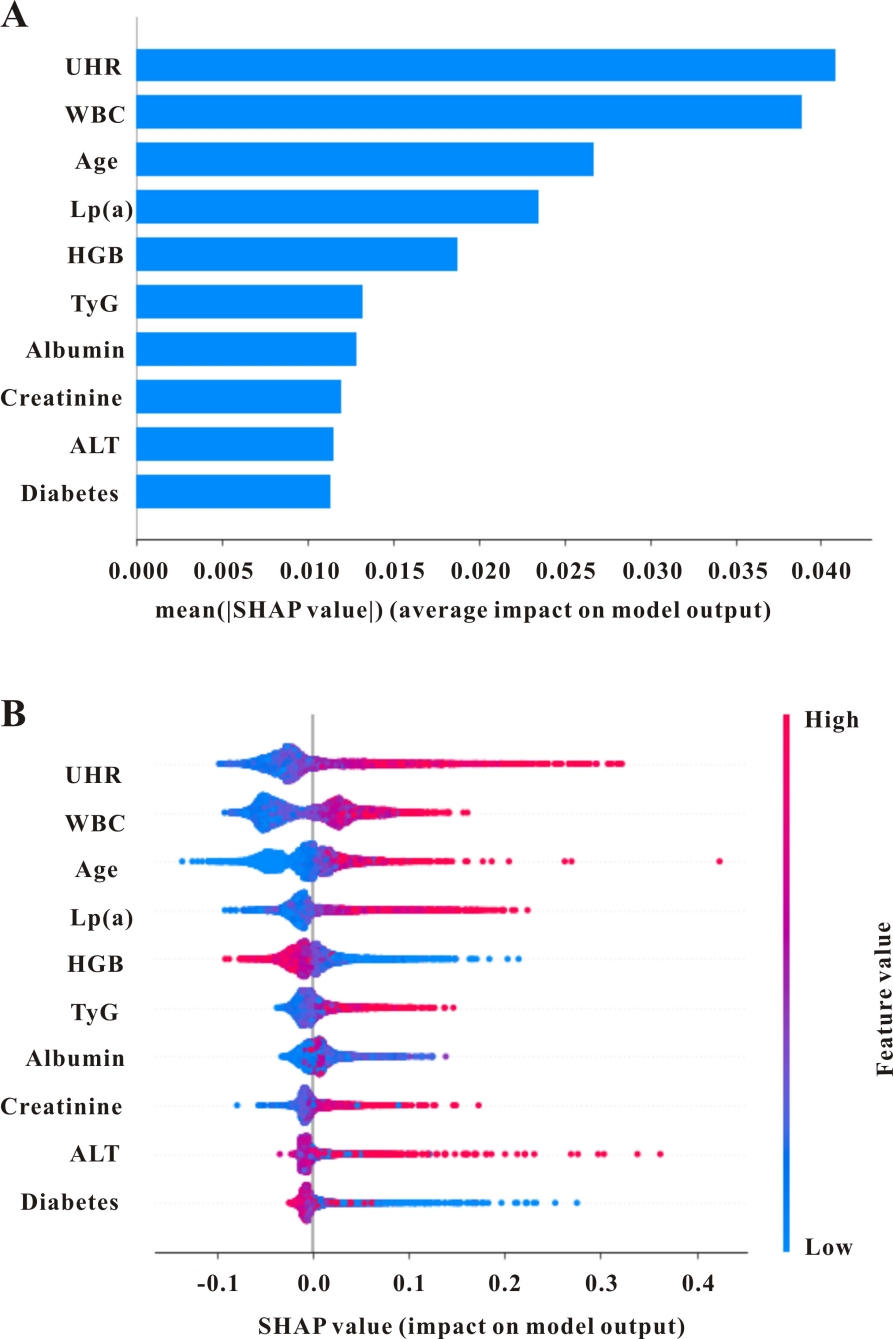
**The global interpretation of the lightGBM model and feature importance**. (A) SHAP summary plot shows the top features of the LightGBM model’s prediction of acute coronary syndromes. Features are positioned along the y-axis based on importance. (B) SHAP summary plot of the features of the LightGBM model. A higher SHAP value for a feature indicates a greater likelihood of in-hospital mortality. Red denotes higher feature values, while blue indicates lower ones. SHAP, SHapley Additive exPlanations; WBC, white blood cells; ALT, alanine aminotransferase; UHR, uric acid to high-density lipoprotein-cholesterol ratio; TyG, triglyceride-to-glucose index; HGB, hemoglobin; Lp(a), lipoprotein(a).

The global explainability of the LightGBM model, along with the ten most predominant factors, is displayed in the SHAP summary plot (Fig. 2). Factors such as age, history of diabetes, white blood cell (WBC) count, hemoglobin, alanine aminotransferase (ALT), albumin (ALB), creatinine, TyG index, UHR, and lipoprotein(a) (Lp(a)) were the principal elements influencing the model’s final outcome. Therefore, we employed the cutoff points derived from the ROC curves and clinical judgment on the training set for model simplification. These cutoffs were used to categorize the variables into two groups each: age (<60 vs. ≥60), WBC (<6.8 vs. ≥6.8), ALT (<26 vs. ≥26), ALB (<38.3 vs. ≥38.3), creatinine (<71.3 vs. ≥71.3), Lp(a) (<19.5 vs. ≥19.5), UHR (<263.0 vs. ≥263.0), and TyG index (<8.9 vs. ≥8.9).

**Fig. 3.**
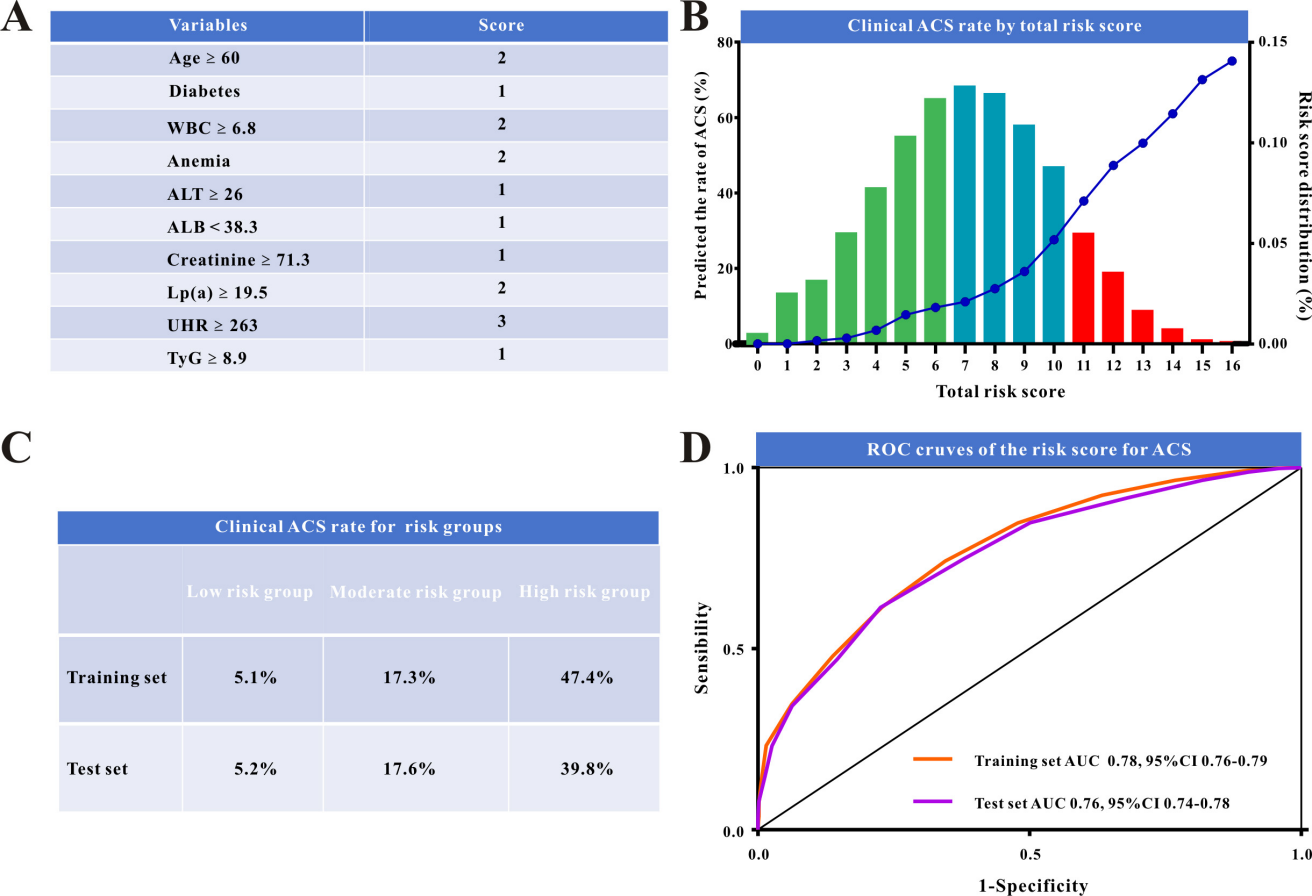
**The risk model and model evaluation**. (A) Novel risk scoring system for acute coronary syndromes (ACS) in hospitalized patients. (B) Clinical ACS rate by novel risk score plot. (C) Clinical ACS rate for the low-risk, moderate-risk, and high-risk groups. (D) ROC curves comparing the performance of the risk model in the training and in the validation set. ROC, receiver operating characteristic; WBC, white blood cells; ALT, alanine aminotransferase; UHR, uric acid to high-density lipoprotein-cholesterol ratio; TyG, triglyceride-to-glucose index; ALB, albumin; AUC, the area under the curve; Lp(a), lipoprotein(a).

Using the coefficients from the LightGBM, the scores for each variable were determined using the scoring system illustrated in Fig. 3 and explained in Table 3 and **Supplementary Table 2**. The scoring was as follows: age >60 years, 2 points; patients with diabetes, 1 point; baseline WBC ≥6.8 and baseline Lp(a) ≥19.5, 2 points; baseline ALT ≥26, baseline ALB <38.3, baseline creatinine ≥71.3, and baseline TyG index ≥8.9, 1 point; baseline UHR ≥263.0, 3 points; patients with anemia, 2 points.

**Table 3.** **Derived score of the final logistic prediction model for ACS**.

Variables	VIF	β	OR (95% CI)	*p* value	Score
Age ≥60	1.22	0.645	1.91 (1.62–2.24)	<0.001	2
Diabetes	1.18	0.153	1.17 (1.01–1.36)	0.045	1
WBC ≥6.8	1.01	0.817	2.27 (1.97–2.60)	<0.001	2
Anemia	1.31	0.516	1.68 (1.43–1.97)	<0.001	2
ALT ≥26	1.02	0.323	1.38 (1.16–1.64)	<0.001	1
ALB <38.3	1.24	–0.339	0.71 (0.62–0.82)	<0.001	1
Creatinine ≥71.3	1.28	0.362	1.44 (1.23–1.67)	<0.001	1
Lp(a) ≥19.5	1.09	0.764	2.15 (1.75–2.64)	<0.001	2
UHR ≥263	1.17	1.184	3.27 (2.83–3.78)	<0.001	3
TyG ≥8.9	1.27	0.307	1.36 (1.17–1.58)	<0.001	1

ACS, acute coronary syndrome; OR, odds ratio; 95% CI, 95% confidence interval; VIF, variance inflation factor; WBC, white blood cells; ALT, alanine aminotransferase; ALB, albumin; UHR, uric acid to high-density lipoprotein-cholesterol ratio; TyG, triglyceride-to-glucose index.

These scores were used to calculate the overall risk score for each patient (Fig. 3A). The model performed well in risk stratification, indicating a proportionate increase in the incidence of ACS with higher risk scores (Fig. 3B). We observed distinct ACS rates across six risk score categories and categorized patients into low-risk (n = 3014), intermediate-risk (n = 3209), and high-risk (n = 865) groups depending on their related hazards. Scores ≤6 were classified as low-risk, with ACS rates below 10%. Scores ≥11 were classified as high-risk, with ACS rates exceeding 40%. Fig. 3B depicts the intermediate-risk group with scores ranging from 7 to 10. **Supplementary Table 3** summarizes the baseline characteristics of each risk category. **Supplementary Fig. 3** showed the calibration curves and decision curve analysis of the risk score for ACS in both sets. The risk-stratification model predicted ACS rates of 5.1% in the low-risk group, 17.3% in the intermediate-risk group, and 47.4% in the high-risk group in the training sample. Furthermore, identical findings were observed in the test set (Fig. 3C). The AUCs for ACS prediction were 0.78 and 0.76 in the training and validation sets, respectively (Fig. 3D). Logistic regression analysis showed that high-risk patients had a 16-fold higher incidence of ACS than low-risk patients (odds ratio [OR]: 16.7; 95% confidence interval [CI]: 13.6–20.6; *p* < 0.001). Intermediate-risk patients had a nearly 4-fold higher incidence of ACS than low-risk patients (OR: 3.88; 95% CI: 3.22–4.68; *p* < 0.001).

The model’s ability to independently stratify risk within cohort subgroups was evaluated using a subgroup analysis for each variable. The model performed well across these subgroups, with full findings provided in **Supplementary Tables 3,4**.

## 4. Discussion

In this study, we developed and evaluated an explainable prediction model based on lipid-related indicators to predict ACS in hospitalized patients. Using data from a large multicenter study, we demonstrated that our risk stratification score achieved considerable accuracy in predicting ACS. To the best of our knowledge, this is the first and most extensive study to use both conventional and ML approaches for this therapeutic assignment. Furthermore, our study had the largest sample size to date, encompassing over 10,000 hospitalized patients from three major hospitals.

ACS is a complex cardiovascular disease with multifactorial pathogenesis, and lipid abnormalities are among the most critical risk factors [24]. Traditionally, LDL-C has been the primary target of lipid-lowering therapy and is regarded as the “gold standard” for assessing cardiovascular risk [25, 26]. However, despite achieving the target LDL-C levels in recent years, the incidence of ACS remains high, suggesting that relying solely on LDL-C may be insufficient to fully capture ACS risk. Thus, identifying new lipid metabolic markers to improve the prediction of ACS has become a priority.

In this study, we systematically explored the role of two emerging lipid-related markers, UHR and TyG, in predicting ACS risk while validating the established role of Lp(a). Incorporating these markers into predictive models showed that UHR and TyG significantly enhanced the performance of the models, underscoring their clinical value in assessing ACS risk. UHR, a novel composite metabolic marker representing the uric acid–to–HDL ratio, has been used to evaluate metabolic health [27]. Elevated uric acid levels are strongly associated with various metabolic disorders such as hypertension, obesity, and diabetes [28, 29], whereas HDL-C is known for its protective cardiovascular effects [30]. Our findings showed that higher UHR values were strongly associated with an increased risk of ACS, suggesting that UHR not only reflects lipid metabolism but may also interact with other risk factors, such as metabolic syndrome, to serve as an effective tool for cardiovascular risk assessment.

Lp(a) is a lipoprotein particle structurally similar to LDL-C but contains a unique protein component, apolipoprotein(a), which has long been recognized as an independent risk factor for atherosclerosis and thrombosis [31, 32]. Our findings further support its role in ACS, with elevated Lp(a) levels significantly associated with higher ACS risk. However, the primary focus of our study was TyG, an indicator of insulin resistance that combines triglyceride and fasting glucose levels. Insulin resistance is a pivotal contributor to atherosclerosis and cardiovascular diseases. Our results demonstrated that TyG was strongly associated with ACS occurrence. Given the increasing prevalence of obesity and metabolic syndrome, TyG—a simple and accessible marker—has substantial potential for predicting cardiovascular events. Collectively, UHR, TyG, and Lp(a) offer valuable insights for improving ACS risk assessment in clinical practice.

We employed several machine learning models to evaluate the predictive effects of these novel lipid markers. Compared with traditional models, the Random Forest and LightGBM models significantly improved the prediction accuracy after incorporating UHR, Lp(a), and TyG. This improvement reflects a substantial enhancement in the predictive power from including these markers. Notably, LightGBM outperformed the other models in terms of sensitivity and accuracy, demonstrating its strength in handling complex, high-dimensional data. This data-driven ML approach offers new possibilities for the early detection of cardiovascular diseases. This enables a clearer understanding of the relative contributions of various risk factors to disease progression. More importantly, an easy-to-use risk score based on the top 10 features derived from the ML models performed relatively well in both the training and validation sets. Patients in the high-risk group had the highest likelihood for ACS, and this trend remained robust across all patients, regardless of comorbidities. ML models, which can handle large amounts of data and complex relationships by extracting important metrics, are widely used for early diagnosis and prognosis assessment in patients with ACS. The primary benefits of ML are its strong performance, improved stability in selecting key metrics, and significantly reduced computation time, making it suitable for handling large datasets. Our study involved a concurrent analysis of three hospital databases. Given the large sample size and abundance of clinical variables, ML techniques are optimal for identifying important ACS risk factors in hospitalized patients. Several ACS risk scores have been identified in previous studies; among them, the GRACE and TIMI scores may be the most widely accepted for predicting the incidence and prognosis of ACS. However, their performance varies across studies, particularly in China. Using data from a prospective Chinese registry, Wang *et al*. [33] reported that the Biomarker-based Prognostic Assessment for Patients with Stable Angina and Acute Coronary Syndromes (BIPass) score outperformed the GRACE and TIMI risk scores in predicting cardiovascular events in patients with ACS. Unlike the above-mentioned studies that examined prognosis in patients with ACS, we focused only on the risk of ACS in hospitalized patients and did not explore the association between this risk score and prognosis in all patients. Moreover, our risk score has relatively good predictive power for ACS in both cohorts, enabling all clinicians, not just cardiologists, to identify hospitalized patients at a high risk of ACS using this score.

Another key innovation of this study is its use of a multicenter design with extensive data analysis. We analyzed large-scale datasets from different regions and populations to conduct stratified analyses and validation. This multicenter design improved the external validity and generalizability of the model, ensuring stability and consistency across subgroups and reducing biases caused by regional and population differences. By integrating data from multiple centers, our findings provide a more robust basis for clinical generalization and hold significant potential for widespread application in the global prevention and management of cardiovascular diseases.

This study had several limitations. First, as a retrospective analysis, causality cannot be established between novel lipid-related markers (UHR, Lp(a), TyG) and ACS, only associations. Biases in data collection, differences in participating hospitals, and patient selection may affect the generalizability of the findings, despite the efforts to minimize them through a multicenter analysis. Second, the predictive models may have been overfitted, as indicated by the discrepancy in the AUC values between the training and test sets. Third, we did not compare the performance of our risk score for ACS with other established scores, such as the GRACE and TIMI scores, which were primarily designed for prognostic assessment in confirmed ACS patients. Future prospective studies were advocated to validate our score alongside these tools in a cohort where both diagnostic and prognostic data are available. Moreover, we did not investigate the association between this score and short- or long-term outcomes in patients with ACS. Some traditional risk factors (e.g., smoking, alcohol use, and medical history) were not included in this risk score, which may explain its relatively weaker performance in certain aspects. Furthermore, potential confounders such as lifestyle, medication use, and genetic factors were not fully controlled. Prospective cohort studies are required to validate these markers and confirm their clinical utility.

## 5. Conclusions

Our study effectively designed and internally validated a predictive risk stratification model for ACS in hospitalized patients using lipid-related indicators. By leveraging explainable ML approaches such as SHAP, we gained valuable insights into the correlations between crucial parameters and ACS, while the final model demonstrated strong predictive accuracy. This risk stratification model could help doctors more effectively pinpoint patients at increased risk of ACS, implement suitable preventive measures, and ultimately lower morbidity while improving quality of life. This study demonstrates the prospective relevance of a unique risk stratification model based on lipid-related indicators for developing personalized therapy and improving clinical decision-making for ACS.

## Data Availability

The datasets used and analyzed during the current study are available from the corresponding author upon reasonable request.

## Abbreviations

ACS, acute coronary syndrome; AUC, area under the curve; BIPass, Biomarker-based Prognostic Assessment for Patients with Stable Angina and Acute Coronary Syndromes; GRACE, Global Registry of Acute Coronary Events; HDL-C, high-density lipoprotein cholesterol; LDL-C, low-density lipoprotein cholesterol; LightGBM, Light Gradient Boosting Machine; Lp(a), lipoprotein(a); ML, machine learning; SHAP, SHapley Additive exPlanations; TIMI, thrombolysis in myocardial infarction; TyG, triglyceride-glucose index; UHR, uric acid-to-HDL-C ratio; RC, residual cholesterol; ROC, receiver operating characteristic; WBC, white blood cells; HGB, hemoglobin; ALT, alanine aminotransferase; ALB, albumin.

## Supplementary Material

Supplementary material associated with this article can be found, in the online version, at https://doi.org/10.31083/RCM44578.
