# Supplementary material for: A Novel Risk Score Based on Lipid-Related Biomarkers for Acute Coronary Syndromes: A Multicenter Machine Learning Study
Source: Rev Cardiovasc Med. 2026 Feb 4;27(2):44578. doi: 10.31083/RCM44578 (PMC12960006; doi:10.31083/RCM44578)
Supplement: Supplementary file 1 [file 2153-8174-27-2-44578-s1.zip › Supplementary Material.docx]

**Supplemental methods**

**Feature selection and extraction**: We based our selection criteria on clinical knowledge, findings from earlier research, and the availability of data. Features with less than 20% missing data were kept, and any missing values were filled in using the k-nearest-neighbor method, considering that some models require complete datasets for full functionality. As for lipid-related markers, we firstly considered the most prevalent lipid indicators in the preliminary analysis of some data, such as total cholesterol, triglycerides, HDL-C, LDL-C, apolipoprotein A1, apolipoprotein B, and lipoprotein A (LpA), however, only the LpA performed relatively good. thus, we retrieved previous studies and finally determined to add the following lipid-related indicators (Residual cholesterol, TyG index, uric acid to HDL-C ratio, and LpA) to improve the performance of final model. In the end, our enhanced database included 27 clinical variables, or "features," which served as candidate predictors for training the models.

**Data splitting strategy and model evaluation:** The dataset was split into training (70%) and test (30%) sets. We enhanced the stratification approach and ensured that the training and test sets had similar distributions by using a modified stratified data splitting strategy. We formed a stratification group using the percentiles of event status within each event category. This approach guaranteed that the distribution of event values was consistent between the training and test sets for each event category. The training set was used for model training and optimization (with repeated 5-fold cross validation). The test set was used for model evaluation. The area under the ROC curve (AUROC) was used to measure the overall model’s predictive performance.

**Machine Learning (ML) models**

ML models require fewer assumptions about data distribution and relationships, which makes them ideal for clinical analysis tasks and they are capable of examining numerous potentially relevant covariates and using intricate statistical techniques to develop an optimal risk model. Thus, we applied 8 ML models, Decision Tree, Random Forest, Extreme Gradient Boosting (XGBoost), Multilayer Perceptron (MLP), Light Gradient Boosting Machine (LightGBM), support vector machine (SVM), elastic network (ENet), and Logistic Regression. All of these methods have been proved previously to be capable of handling these data.

**Feature engineering**: We employed SHAP analysis to pinpoint possibly significant variables and explored whether models could effectively forecast outcome or improve predictions by choosing fewer variables. In the process of developing the model, features that had the smallest effect on the outcome were removed one by one, and the models were refitted repeatedly with reduced feature sets. We kept eliminating recursively until we noticed a notable drop in model performance. Furthermore, we used SHAP analysis to determine the best clinical thresholds for specific continuous variables, which helps simplify the model for future use and refines our models by concentrating on the most predictive variables.

**Statistical Analysis:** All analyses and calculations were performed with Python (Version 3.11) and R (version 4.3.2). After identifying the top 10 predictors using the LightGBM model and SHAP analysis, we converted these continuous variables into categorical ones based on optimal cut-off values determined by ROC analysis. A multivariable logistic regression model was then fitted using these categorized variables. The beta coefficients from this model were used to assign points for each variable, proportional to their weighted contribution to ACS risk. The total risk score for each patient was calculated as the sum of these points, and patients were stratified into low-, intermediate-, and high-risk groups based on the score distribution and observed event rates.

The detailed breakdown of the libraries used in our analysis, matched with their respective methods, is as follows:

**Data preprocessing, and visualization:** numpy, pandas, matplotlib, seaborn, sklearn. preprocessing (MinMaxScaler, StandardScaler, OrdinalEncoder, OneHotEncoder).

**Data imputation**: sklearn.impute (KNNImputer).

**Model development**: sklearn.model_selection (RepeatedKFold, train_test_split, cross_val_score), sklearn.ensemble (RandomForestClassifier,), sklearn.svm (SVM), sklearn.linear model (LogisticRegression, ElasticNet), sklearn.tree (DecisionTreeRegressor), skleran.neural network (MLPClassifier), xgboost, lightgbm, shap, sklearn.metrics (roc_curve, auc, roc_auc_score, accuracy_score, precision_score, recall_score, f1_score, confusion_matrix).

**Supplementary Table 1. Baseline characteristics of the training set and the test set.**

| Characteristics | Overall (n=10127) | Training set (n=7088) | Test set (n=3039) | P value |
| --- | --- | --- | --- | --- |
| Age, years old | 64.9 ± 11.1 | 64.9 ± 11.0 | 64.7 ± 11.1 | 0.462 |
| Gender, male, n (%) | 6361 (62.8) | 4466 (63.0) | 1895 (62.4) | 0.534 |
| Current smoker, n (%) | 1701 (16.8) | 1199 (16.9) | 502 (16.5) | 0.624 |
| Comorbidities, n (%) |  |  |  |  |
| Hypertension | 6438 (63.6) | 4472 (36.9) | 1966 (64.7) | 0.125 |
| Diabetes | 2732 (27.0) | 1934 (27.3) | 798 (26.3) | 0.286 |
| HLP | 1647 (16.3) | 1173 (16.5) | 474 (15.6) | 0.234 |
| HUA | 611 (6.0) | 428 (6.0) | 183 (6.0) | 0.974 |
| Stroke | 2691 (26.6) | 1853 (26.1) | 838 (27.6) | 0.135 |
| AF | 1749 (17.3) | 1217 (17.2) | 532 (17.5) | 0.682 |
| Laboratory results |  |  |  |  |
| WBC, × 10^9^/L | 6.6 ± 2.2 | 6.7 ± 2.1 | 6.6 ± 2.2 | 0.644 |
| Hemoglobin, g/L | 137.3 ± 18.2 | 137.2 ± 18.3 | 137.5 ± 18.0 | 0.412 |
| Platelet, × 10^9^/L | 207.3 ± 50.9 | 206.6 ± 50.2 | 208.8 ± 52.4 | 0.101 |
| Lymphocytes, × 10^9^/L | 1.2 (0.5, 1.7) | 1.2 (0.5, 1.7) | 1.2 (0.5, 1.7) | 0.657 |
| Monocytes, × 10^9^/L | 0.5 (0.3, 1.1) | 0.5 (0.3, 1.1) | 0.5 (0.3, 1.0) | 0.857 |
| AST, U/L | 20.0 (14.0, 29.0) | 20.0 (14.0, 29.0) | 20.0 (14.0, 30.0) | 0.584 |
| ALT, U/L | 19.0 (13.0, 28.0) | 19.0 (13.0, 28.0) | 19.0 (13.0, 28.0) | 0.784 |
| TBIL, umol/L | 11.4 (8.5, 15.1) | 11.4 (8.5, 15.1) | 11.2 (8.7, 14.9) | 0.488 |
| DBIL, umol/L | 3.3 (2.4, 4.6) | 3.3 (2.4, 4.6) | 3.3 (2.4, 4.4) | 0.348 |
| Albumin, g/L | 39.9 ± 4.3 | 39.9 ± 4.3 | 40.0 ± 4.3 | 0.274 |
| BUN, umol/L | 5.0 (4.2, 5.9) | 5.0 (4.2, 5.9) | 5.0 (4.2, 5.9) | 0.182 |
| Creatinine, umol/L | 84.6 ± 25.4 | 84.8 ± 25.5 | 84.2 ± 25.2 | 0.674 |
| Potassium, | 4.0 ± 0.4 | 4.0 ± 0.4 | 4.0 ± 0.4 | 0.154 |
| Sodium | 138.3 ± 3.8 | 138.3 ± 3.7 | 138.3 ± 3.8 | 0.717 |
| Lipid-related biomarkers |  |  |  |  |
| RC | 0.5 (0.3, 0.7) | 0.5 (0.3, 0.7) | 0.5 (0.3, 0.7) | 0.054 |
| UHR | 285.4 ± 60.8 | 286.1 ± 60.3 | 283.7 ± 61.9 | 0.478 |
| TyG | 8.8 ± 0.7 | 8.8 ± 0.7 | 8.8 ± 0.7 | 0.925 |
| Lp (A) | 170.5 ± 62.8 | 170.3 ± 61.0 | 170.9 ± 62.8 | 0.915 |
| Clinical outcome |  |  |  |  |
| ACS, n (%) | 1580 (15.6) | 1119 (15.8) | 461 (15.2) | 0.432 |

HLP, hyperlipidemia, HUA, hyperuricemia, AF, atrial fibrillation, WBC, white blood cells, AST, aspartate aminotransferase, ALT, alanine aminotransferase, TBIL, total bilirubin, DBIL, direct bilirubin, BUN, blood urea nitrogen, RC, Residual cholesterol, UHR, Uric acid to high-density lipoprotein-cholesterol ratio, TyG, triglyceride-to-glucose index, ACS, acute coronary syndrome.

**Supplementary Table 2. Baseline characteristics of the risk groups in the training set.**

| Characteristics | Low risk group | Moderate risk group | High risk group | P value |
| --- | --- | --- | --- | --- |
| N | 3014 | 3209 | 865 |  |
| Age, years old | 62.5 ± 10.5 | 66.4 ± 11.1 | 68.8 ± 10.9 | <0.001 |
| Gender, male, n (%) | 1593 (52.9) | 2236 (69.7) | 637 (73.6) | <0.001 |
| Current smoker, n (%) | 402 (13.3) | 620 (19.3) | 177 (20.5) | <0.001 |
| Comorbidities, n (%) |  |  |  |  |
| Hypertension | 1545 (51.3) | 2278 (71.0) | 649 (75.0) | <0.001 |
| Diabetes | 391 (13.0) | 1081 (33.7) | 462 (53.4) | <0.001 |
| HLP | 586 (19.4) | 476 (14.8) | 111 (12.8) | <0.001 |
| HUA | 172 (5.7) | 202 (6.3) | 54 (6.2) | 0.601 |
| Stroke | 554 (18.4) | 1030 (32.1) | 269 (31.1) | <0.001 |
| AF | 682 (22.6) | 441 (13.7) | 94 (10.9) | <0.001 |
| Laboratory results |  |  |  |  |
| WBC, × 10^9^/L | 5.9 ± 2.0 | 7.0 ± 2.6 | 8.0 ± 2.7 | <0.001 |
| Hemoglobin, g/L | 139.1 ± 15.4 | 137.0 ± 19.3 | 131.2 ± 22.1 | <0.001 |
| Platelet, × 10^9^/L | 204.5 ± 56.7 | 207.7 ± 61.7 | 210.0 ± 66.3 | 0.027 |
| Lymphocytes, × 10^9^/L | 0.6 (0.3, 1.5) | 1.3 (0.9, 1.8) | 1.4 (1.0, 1.9) | <0.001 |
| Monocytes, × 10^9^/L | 0.8 (0.4, 0.9) | 0.4 (0.3, 0.6) | 0.5 (0.4, 0.6) | <0.001 |
| AST, U/L | 19.0 (14.0, 29.0) | 20.0 (15.0, 30.0) | 20.0 (14.0, 29.0) | 0.034 |
| ALT, U/L | 17.0 (13.0, 22.0) | 20.0 (14.0, 30.0) | 31.0 (19.0, 44.0) | <0.001 |
| TBIL, umol/L | 11.6 (8.8, 15.4) | 11.3 (8.4, 15.0) | 11.0 (8.0, 15.1) | 0.410 |
| DBIL, umol/L | 3.1 (2.3, 4.2) | 3.5 (2.5, 4.8) | 3.6 (2.4, 5.0) | <0.001 |
| Albumin, g/L | 40.1 ± 4.3 | 39.9 ± 4.3 | 39.4 ± 4.4 | <0.001 |
| BUN, umol/L | 5.1 (4.3, 6.0) | 5.0 (4.2, 5.9) | 5.0 (4.3, 5.9) | 0.012 |
| Creatinine, umol/L | 72.2 ± 20.2 | 89.7 ± 20.4 | 110.3 ± 29.2 | <0.001 |
| Potassium, | 4.0 ± 0.4 | 4.0 ± 0.4 | 4.0 ± 0.5 | <0.001 |
| Sodium | 138.3 ± 3.8 | 138.4 ± 3.6 | 138.4 ± 3.7 | 0.499 |
| Lipid-related biomarkers |  |  |  |  |
| RC | 0.5 (0.3, 0.7) | 0.5 (0.3, 0.8) | 0.5 (0.3, 0.8) | <0.001 |
| UHR | 205.7 ± 56.9 | 331.6 ± 72.4 | 397.5 ± 110.3 | <0.001 |
| TyG | 8.7 ± 0.6 | 8.9 ± 0.7 | 9.1 ± 0.7 | <0.001 |
| Lp (A) | 111.2 ± 33.9 | 203.7 ± 46.4 | 252.5 ± 57.9 | <0.001 |
| Total risk score | 4.3 ± 1.6 | 8.4 ± 1.1 | 11.9 ± 1.1 | <0.001 |
| Clinical outcome |  |  |  |  |
| ACS, n (%) | 154 (5.1) | 555 (17.3) | 410 (47.4) | <0.001 |

HLP, hyperlipidemia, HUA, hyperuricemia, AF, atrial fibrillation, WBC, white blood cells, AST, aspartate aminotransferase, ALT, alanine aminotransferase, TBIL, total bilirubin, DBIL, direct bilirubin, BUN, blood urea nitrogen, RC, Residual cholesterol, UHR, Uric acid to high-density lipoprotein-cholesterol ratio, TyG, triglyceride-to-glucose index, ACS, acute coronary syndrome.

**Supplementary Table 3. Logistic analysis of risk score for ACS in the training set.**

| Subgroups | Case/N | OR (95%CI) | P value^1^ |
| --- | --- | --- | --- |
| All |  |  |  |
| Low risk | 154/3014 | Ref. |  |
| Moderate risk | 555/3209 | 3.88 (3.22-4.68) | <0.001 |
| High risk | 410/865 | 16.74 (13.57-20.65) | <0.001 |
| Age |  |  |  |
| < 60 years old |  |  |  |
| Low risk | 63/1687 | Ref. |  |
| Moderate risk | 164/981 | 5.17 (3.82-7.00) | <0.001 |
| High risk | 80/164 | 24.55 (16.52-36.49) | <0.001 |
| ≥ 60 years old |  |  |  |
| Low risk | 91/1327 | Ref. |  |
| Moderate risk | 391/2228 | 2.89 (2.28-3.67) | <0.001 |
| High risk | 330/701 | 12.08 (9.32-15.66) | <0.001 |
| Gender |  |  |  |
| Male |  |  |  |
| Low risk | 108/1593 | Ref. |  |
| Moderate risk | 437/2236 | 3.34 (2.68-4.17) | <0.001 |
| High risk | 319/637 | 13.79 (10.75-17.70) | <0.001 |
| Female |  |  |  |
| Low risk | 46/1421 | Ref. |  |
| Moderate risk | 118/973 | 4.13 (2.90-5.86) | <0.001 |
| High risk | 91/228 | 19.86 (13.34-29.49) | <0.001 |
| Hypertension |  |  |  |
| No |  |  |  |
| Low risk | 60/1469 | Ref. |  |
| Moderate risk | 153/931 | 4.62 (3.38-6.30) | <0.001 |
| High risk | 131/216 | 36.19 (24.85-52.70) | <0.001 |
| Yes |  |  |  |
| Low risk | 94/1545 | Ref. |  |
| Moderate risk | 402/2278 | 3.31 (2.62-4.18) | <0.001 |
| High risk | 279/649 | 11.64 (8.97-15.10) | <0.001 |
| Diabetes mellitus |  |  |  |
| No |  |  |  |
| Low risk | 125/2623 | Ref. |  |
| Moderate risk | 370/2128 | 4.21 (3.40-5.20) | <0.001 |
| High risk | 213/403 | 22.40 (17.18-29.22) | <0.001 |
| Yes |  |  |  |
| Low risk | 29/391 | Ref. |  |
| Moderate risk | 185/1081 | 2.58 (1.71-3.88) | <0.001 |
| High risk | 197/462 | 9.28 (6.09-14.14) | <0.001 |
| HLP |  |  |  |
| No |  |  |  |
| Low risk | 123/2428 | Ref. |  |
| Moderate risk | 483/2733 | 4.02 (3.27-4.95) | <0.001 |
| High risk | 355/754 | 16.67 (13.24-21.01) | <0.001 |
| Yes |  |  |  |
| Low risk | 31/586 | Ref. |  |
| Moderate risk | 72/476 | 3.19 (2.06-4.96) | <0.001 |
| High risk | 55/111 | 17.58 (10.47-29.54) | <0.001 |
| Atrial fibrillation |  |  |  |
| No |  |  |  |
| Low risk | 110/2332 | Ref. |  |
| Moderate risk | 493/2768 | 4.38 (3.53-5.43) | <0.001 |
| High risk | 364/771 | 18.07 (14.24-22.92) | <0.001 |
| Yes |  |  |  |
| Low risk | 44/682 | Ref. |  |
| Moderate risk | 62/441 | 2.37 (1.58-3.56) | <0.001 |
| High risk | 46/94 | 13.90 (8.37-23.07) | <0.001 |

^1^The P-value is corrected by the method of Bonferroni for multiple testing at α= 0.05/6 significance level. HLP, hyperlipidemia, AF, atrial fibrillation, OR, odds ratio, 95%CI, 95% confidence index.

**Supplementary Table 4. Logistic analysis of risk score for ACS in the test set.**

| Subgroups | Case/N | OR (95%CI) | P value^1^ |
| --- | --- | --- | --- |
| All |  |  |  |
| Low risk | 67/1278 | Ref. |  |
| Moderate risk | 246/1397 | 3.86 (2.91-5.12) | <0.001 |
| High risk | 148/364 | 12.38 (9.97-17.10) | <0.001 |
| Age |  |  |  |
| < 60 years old |  |  |  |
| Low risk | 28/701 | Ref. |  |
| Moderate risk | 90/463 | 5.80 (3.73-9.03) | <0.001 |
| High risk | 30/74 | 16.39 (9.00-29.82) | <0.001 |
| ≥ 60 years old |  |  |  |
| Low risk | 39/577 | Ref. |  |
| Moderate risk | 156/934 | 2.77 (1.92-4.00) | <0.001 |
| High risk | 118/290 | 9.46 (6.34-14.13) | <0.001 |
| Gender |  |  |  |
| Male |  |  |  |
| Low risk | 53/655 | Ref. |  |
| Moderate risk | 193/963 | 2.85 (2.06-3.93) | <0.001 |
| High risk | 118/277 | 8.43 (5.83-12.18) | <0.001 |
| Female |  |  |  |
| Low risk | 14/623 | Ref. |  |
| Moderate risk | 53/434 | 6.05 (3.31-11.06) | <0.001 |
| High risk | 30/87 | 22.90 (11.48-45.65) | <0.001 |
| Hypertension |  |  |  |
| No |  |  |  |
| Low risk | 34/590 | Ref. |  |
| Moderate risk | 76/390 | 3.96 (2.58-6.07) | <0.001 |
| High risk | 54/93 | 22.64 (13.22-38.78) | <0.001 |
| Yes |  |  |  |
| Low risk | 33/688 | Ref. |  |
| Moderate risk | 170/1007 | 4.03 (2.74-5.93) | <0.001 |
| High risk | 94/271 | 10.54 (6.86-16.20) | <0.001 |
| Diabetes mellitus |  |  |  |
| No |  |  |  |
| Low risk | 53/1096 | Ref. |  |
| Moderate risk | 180/965 | 4.51 (3.28-6.21) | <0.001 |
| High risk | 82/180 | 16.47 (11.01-24.63) | <0.001 |
| Yes |  |  |  |
| Low risk | 14/182 | Ref. |  |
| Moderate risk | 66/432 | 2.16 (1.18-3.96) | <0.001 |
| High risk | 66/184 | 6.71 (3.61-12.51) | <0.001 |
| HLP |  |  |  |
| No |  |  |  |
| Low risk | 52/1047 | Ref. |  |
| Moderate risk | 222/1195 | 4.37 (3.19-5.98) | <0.001 |
| High risk | 52/1047 | 13.22 (9.26-18.88) | <0.001 |
| Yes |  |  |  |
| Low risk | 15/231 | Ref. |  |
| Moderate risk | 24/202 | 1.94 (1.19-3.81) | <0.001 |
| High risk | 16/41 | 9.22 (4.07-20.86) | <0.001 |
| Atrial fibrillation |  |  |  |
| No |  |  |  |
| Low risk | 52/988 | Ref. |  |
| Moderate risk | 210/1182 | 3.89 (2.83-5.34) | <0.001 |
| High risk | 136/337 | 12.18 (8.55-17.35) | <0.001 |
| Yes |  |  |  |
| Low risk | 15/290 | Ref. |  |
| Moderate risk | 36/215 | 3.69 (1.96-6.93) | <0.001 |
| High risk | 12/27 | 14.67 (5.85-36.80) | <0.001 |

^1^The P-value is corrected by the method of Bonferroni for multiple testing at α= 0.05/6 significance level. HLP, hyperlipidemia, AF, atrial fibrillation, OR, odds ratio, 95%CI, 95% confidence index.


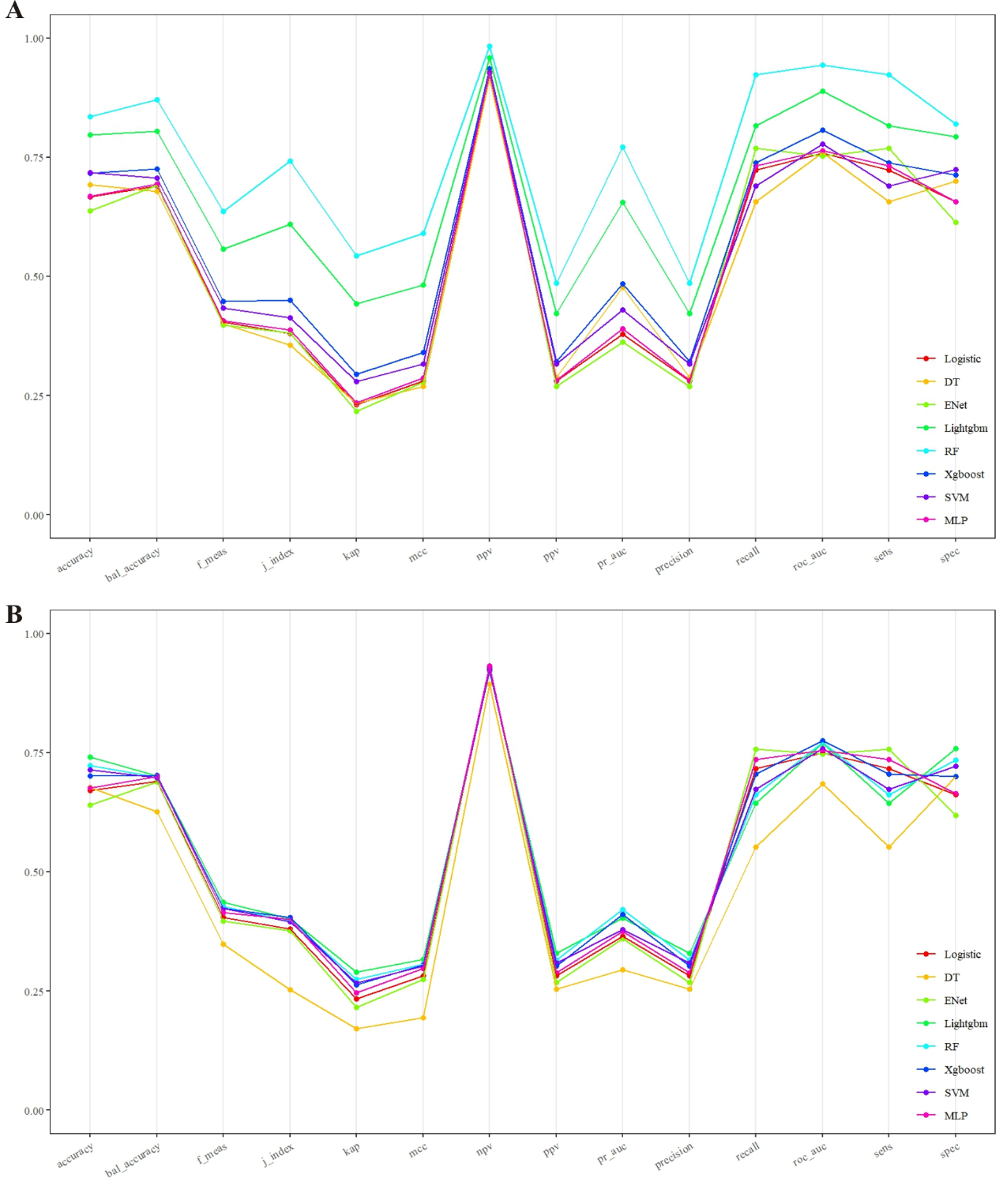


**Supplementary Fig. 1.** The performance of different machine learning models across key metrics in training set (A) and in the validation set (B).


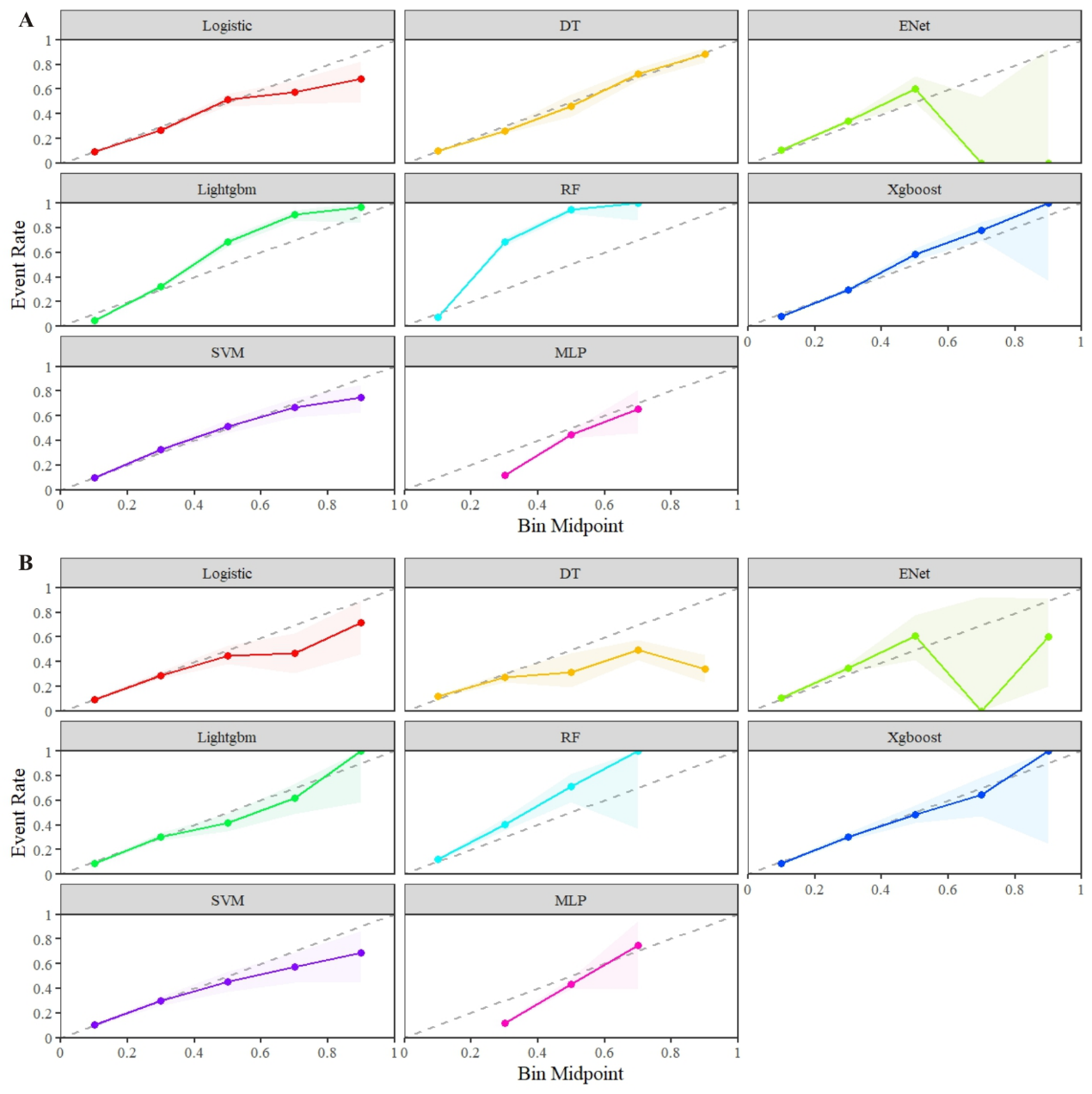


**Supplementary Fig. 2.** The calibration curves of different machine learning models in training set (A) and in the validation set (B).


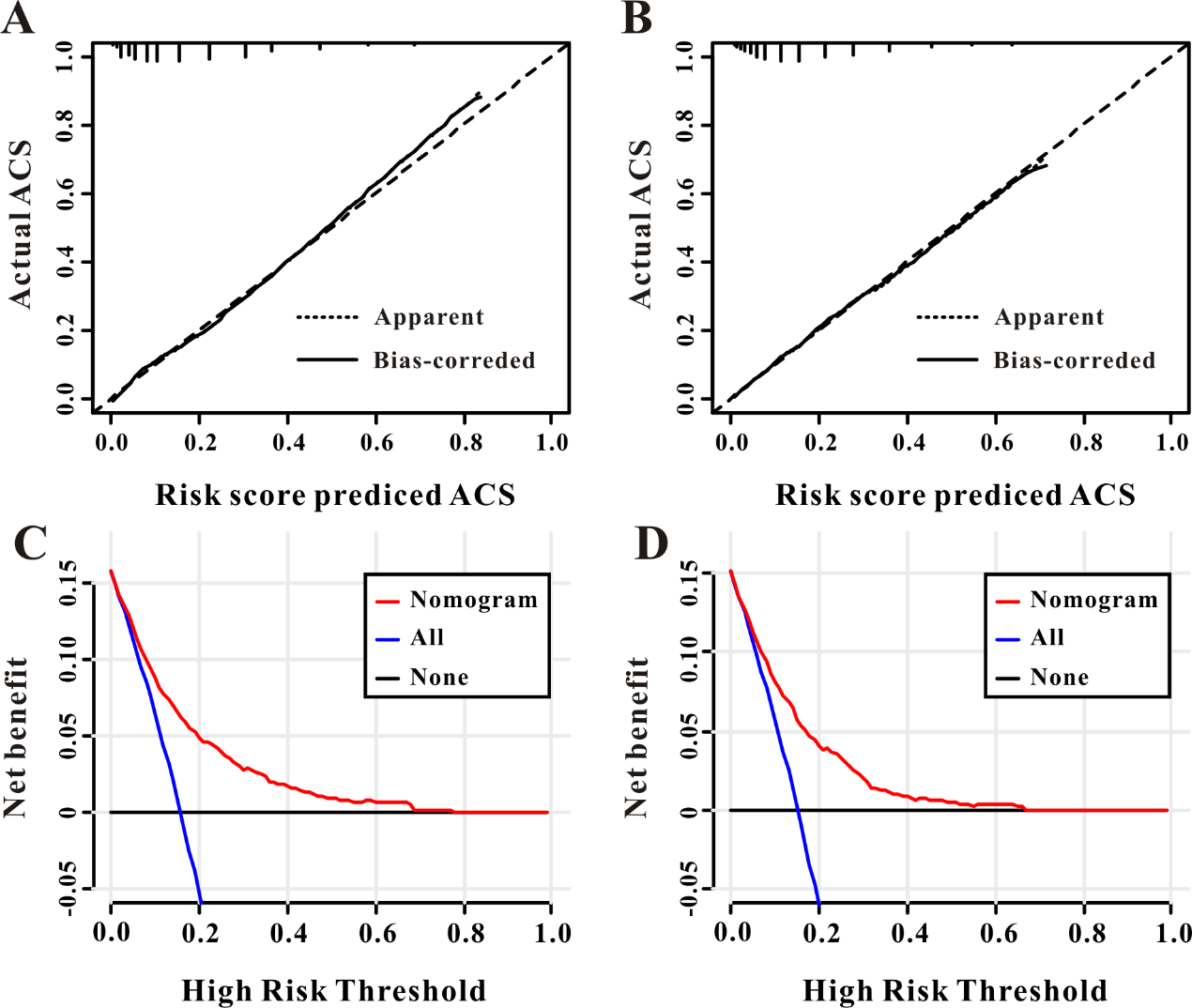


**Supplementary Fig. 3.** The calibration curves of the risk score for ACS in the training set (A) and the validation set (B). Decision curve analysis of the risk score for ACS in the training (C) and the validation cohorts (D).
